# Using human-centered design to co-design dedicated menstrual health spaces with people who menstruate in Bidi Bidi refugee settlement, Uganda: Learnings for further adaptation and scale in humanitarian settings

**DOI:** 10.1186/s12905-023-02421-0

**Published:** 2023-06-20

**Authors:** Aly Beeman, Joseph Kwesiga, Nicole Ippoliti, Tanya Bhandari, Geetika Pandya, Faith Anne Acam, Saehee Lee, Rebecca Hope, Theodora Gibbs, Jordan Levine

**Affiliations:** Youth Development Labs (YLabs), KN 14 St, Kigali, Rwanda

**Keywords:** Menstruation, Menstrual hygiene management, Gender, Refugee, Water, sanitation and hygiene (WASH), Waste management, Human-centered design

## Abstract

**Background:**

Many people who menstruate in low- and middle-income countries struggle to manage their menstruation safely, hygienically, and with dignity. This is exacerbated in humanitarian settings with limited access to menstrual products and safe, private spaces for changing, washing, and disposing of menstrual products. To address these challenges, Youth Development Labs (YLabs) used a human-centered design approach to co-design the Cocoon Mini, a safe, physical structure for managing menstruation in the Bidi Bidi Refugee Settlement in Uganda.

**Methods:**

The study comprised five phases, including background research, design research, rough prototyping, live prototyping, and a pilot study. A total of 340 people, including people who menstruate, male community members, and community stakeholders, participated in interviews, focus groups, and co-design sessions. Solution prototypes were created, evaluated, and iterated upon in each successive project phase. The final intervention design, the Cocoon Mini, was evaluated qualitatively for feasibility and acceptability during a three-month pilot using structured interviews with 109 people who menstruate utilizing Cocoon Mini structures, 64 other community members, and 20 Cocoon Mini supervisors.

**Results:**

Results showed widespread desirability and acceptability of the Cocoon Mini among people who menstruate and other community members. Overall, 95% (104/109) of people who menstruate stated the space had made menstrual health management easier, primarily by providing designated waste bins, solar lights, and additional water sources. The Cocoon Mini provided an increased sense of physical and psychological safety in knowing where to privately manage menstruation. Furthermore, the Cocoon Mini demonstrated that an intervention could be run and maintained sustainably at the household level in humanitarian contexts, without continued external stakeholder intervention. Each Cocoon Mini structure costs approximately $360 USD to build and maintain and serves 15–20 people who menstruate, leading to a cost per person of $18-$24. Furthermore, attaching an incinerator to the structure for easier and quicker disposal of waste bin contents (compared to transporting full waste bins elsewhere) costs $2110 USD.

**Conclusions:**

People who menstruate lack access to safe, private spaces for menstrual health and product disposal in humanitarian settings. The Cocoon Mini provides a solution for the safe and effective management of menstruation. Customizing and scaling up dedicated menstrual health spaces should be considered a high-priority intervention in humanitarian settings.

## Background

Many people who menstruate across low and middle-income countries (LMICs) face frequent challenges managing their menstruation safely, hygienically, and with dignity. With limited access to sanitary pads, washing supplies, and safe, private, physical spaces, menstrual health (MH) remains a pressing problem [1–6]. Furthermore, this issue disproportionately impacts those in humanitarian settings due to both environmental factors (such as water shortages, crowded conditions, and poor facilities) and systemic factors (such as economic hardship and insufficient MH education) [7–9]. MH has historically been an overlooked component in both acute and protracted emergency situations, as it is not considered immediately life-threatening [9–11].

MH has gained more attention in humanitarian settings in recent years. Several movements have highlighted the need to tackle MH as a human rights and public health issue critical to achieving the UN’s Sustainable Development Goals [12–14]. However, there is little consensus on what constitutes a comprehensive MH response beyond simple distribution of menstrual products [7]. A comprehensive response may be defined by both ‘hardware’ and ‘software’ solutions [15]. Hardware solutions refer to material and physical interventions, such as building physical infrastructure for privacy and safety, whereas software solutions refer to interventions addressing social stigma and the psychosocial impacts of poor menstrual health [16, 17]. The range of challenges people who menstruate may face in humanitarian settings differs according to the preceding emergency, geographical location, and cultural beliefs about menstruation; therefore, there is a need to tailor programs to the specific contextual nuances [18]. Historically, residents of humanitarian spaces, especially women and girls, have not been extensively engaged or consulted in the design of sanitation facilities, resulting in well-documented shortcomings in the design of services intended for their use [19]. As such, there is an urgent need to actively involve people who menstruate during the design, development, and testing of MH innovations to ensure their needs and preferences are met.

With support from the Office of the Prime Minister and the International Rescue Committee, research and design organization YLabs partnered with Alight, a humanitarian sector non-profit organization, and Kuja Kuja, a global data collection firm, to co-design and evaluate safe menstrual health spaces and services with settlement residents and stakeholders in the Bidi Bidi Refugee Settlement of northwest Uganda. In this paper, we describe how we utilized a five-phase human-centered design (HCD) approach to guide the development of a low-cost, scalable, physical structure for managing menstruation. After designing initial prototypes of these structures, 20 private and accessible MH spaces were built in the settlement during the summer of 2021. Each structure, called a Cocoon Mini (Mini), served between 15 and 20 people who menstruate. A three-month pilot was then conducted to assess the desirability, acceptability, and feasibility of the Mini as an intervention to support safer, more dignified MH practices.

## Methods

**Fig. 1:**
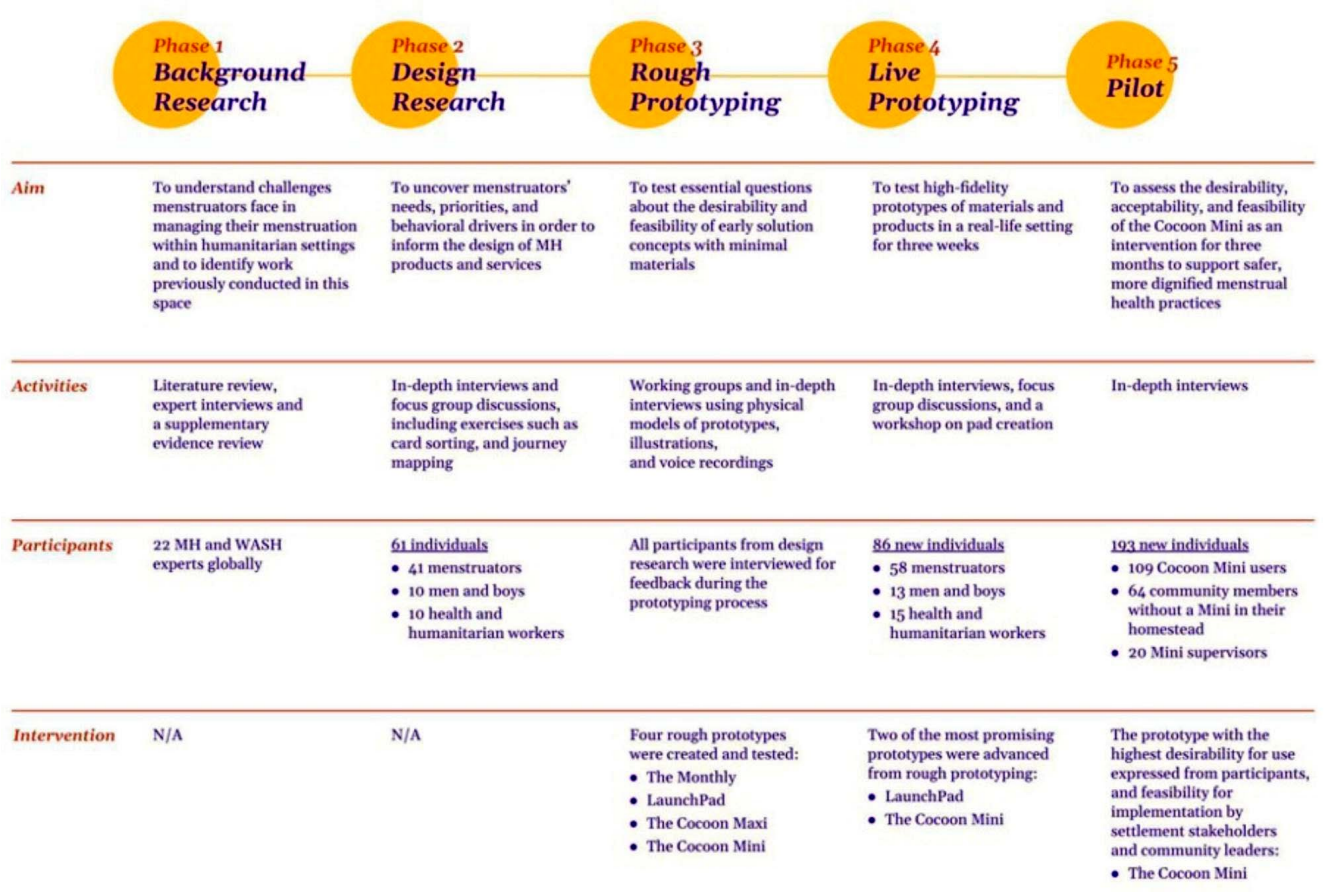
Overview of Phases of HCD Phases and Study Approach

This project consisted of five phases to research, co-design, test, and pilot safe spaces and services for managing menstruation among displaced populations. These phases included: (1) Background Research, (2) Design Research, (3) Rough Prototyping, (4) Live Prototyping, and (5) a three-month Pilot (Fig. 1).

We employed HCD methods to gain comprehensive user insights about participants’ knowledge, attitudes, and perspectives on MH. HCD is a creative, iterative, and participatory innovation process that seeks to engage participants in co-designing, developing, and testing potential solutions [20, 21]. Co-design empowers participants to tailor interventions to their specific health and cultural needs, making MH interventions more acceptable to users and, therefore, more likely to be adopted and sustained [22, 23]. The approach relies on real-world prototyping and rapid iteration of potential solution ideas based on participant feedback.

HCD emphasizes a process of rapidly co-designing, generating, and testing a range of solution ideas, with specific focus on their desirability and feasibility, with minimal material and monetary investment [20, 21]. Desirability refers to the degree to which users like and commit to utilizing the proposed solution to address a given challenge, while feasibility refers to the ease with which the solution can be administered and sustained over time. Each research phase informs the final design by selecting and refining low-fidelity prototypes until one final high-fidelity intervention is implemented and evaluated during a pilot phase.

### Setting

This study takes place in the Bidi Bidi Refugee Settlement of northwest Uganda. This settlement is split into five zones (Zones 1 through 5) and spans 292 square kilometers in the West Nile area of Uganda in the Yumbe district. The settlement was established in 2016 to accommodate the rapid influx of South Sudanese refugees fleeing violent civil war [24]. As of the end of 2021, the settlement is home to nearly 250,000 people and is the largest refugee settlement in Uganda [25]. This project was conducted in the Ariwa I and Ariwa II village clusters within Zone 5. These sites were selected due to a strong local presence of project partners and a documented lack of appropriate water and sanitation services for residents, particularly people who menstruate. Within Zone 5, 39% of households are not able to collect enough water to meet their needs, 4% of households openly defecate, and 6% do not have access to a designated bathing facility [26].

### Phase one: background research

At the start of 2020, we conducted background research to identify cultural and infrastructural challenges people who menstruate face in managing their menstruation within humanitarian settings. This included an evidence review and interviews with six key stakeholders within the Bidi Bidi Settlement to understand critical challenges associated with implementing MH interventions. Our literature review utilized PubMed and Google Scholar for the search of peer reviewed journal articles. We also searched for grey literature using Google to review reports and news articles. Search terms included keywords and MeSH terms of “menstrual hygiene,” “menstrua*,” “refugee,” “IDP,” “displace*,” “emergenc*,” and “crisis.”

Additionally, a supplementary evidence review was undertaken during the pilot to specifically understand people who menstruate’s behavior and social norms regarding the disposal of menstrual products, as this remains one of the most significant sources of shame and barriers to privately managing menstrual health [1]. We investigated menstrual product disposal preferences and behaviors, as well as the impact of these current disposal methods on the individuals, community, and environment. The supplementary evidence review consisted of a literature review using the same methods described above using the keywords “menstrual waste management”, “management disposal”, “menstrual waste disposal”, and “menstrual hygiene management disposal practice”. In addition, we conducted 22 interviews with MH and water, sanitation and hygiene (WASH) experts globally.

### Phase two: design research

Design research aims to uncover people’s needs, priorities, and behavioral drivers in order to inform the design of products and services. We sought to understand the cultural context surrounding menstruation, the challenges of daily life in the settlement that impact MH, and the most desired components of an intervention to improve MH. In September 2020, the YLabs and Kuja Kuja teams conducted design research in the Bidi Bidi Settlement with 61 community members, of which 41 were people who menstruate between the ages of 15–24, 10 were men and boys, and 10 were health and humanitarian workers. We used interviews, focus groups, and activities such as card sorting and journey mapping to better understand people who menstruate’s support networks and available resources [27]. Card sorting involves asking participants to rank a set of choices from most to least important (in this case, potential confidants from whom menstrual hygiene management can be learned). Journey mapping is an activity where participants describe in detail their experience addressing a challenging situation (in this case, one 24-hour period spent managing menstruation) to better elucidate barriers to access. We asked participants to rank the most significant barriers to menstrual health management, including lack of space for drying sanitary products, water for cleaning, access to sanitary products, private spaces for changing, and disposal areas for sanitary products.

During the design research phase, Kuja Kuja research team members living in the community started actively engaging with community leaders and members by hosting sensitization and education meetings about MH to incentivize community buy-in for the project. The sensitization and education meetings helped build community trust and partnership and continued throughout the project.

### Phase three: rough prototyping

**Fig. 2:**
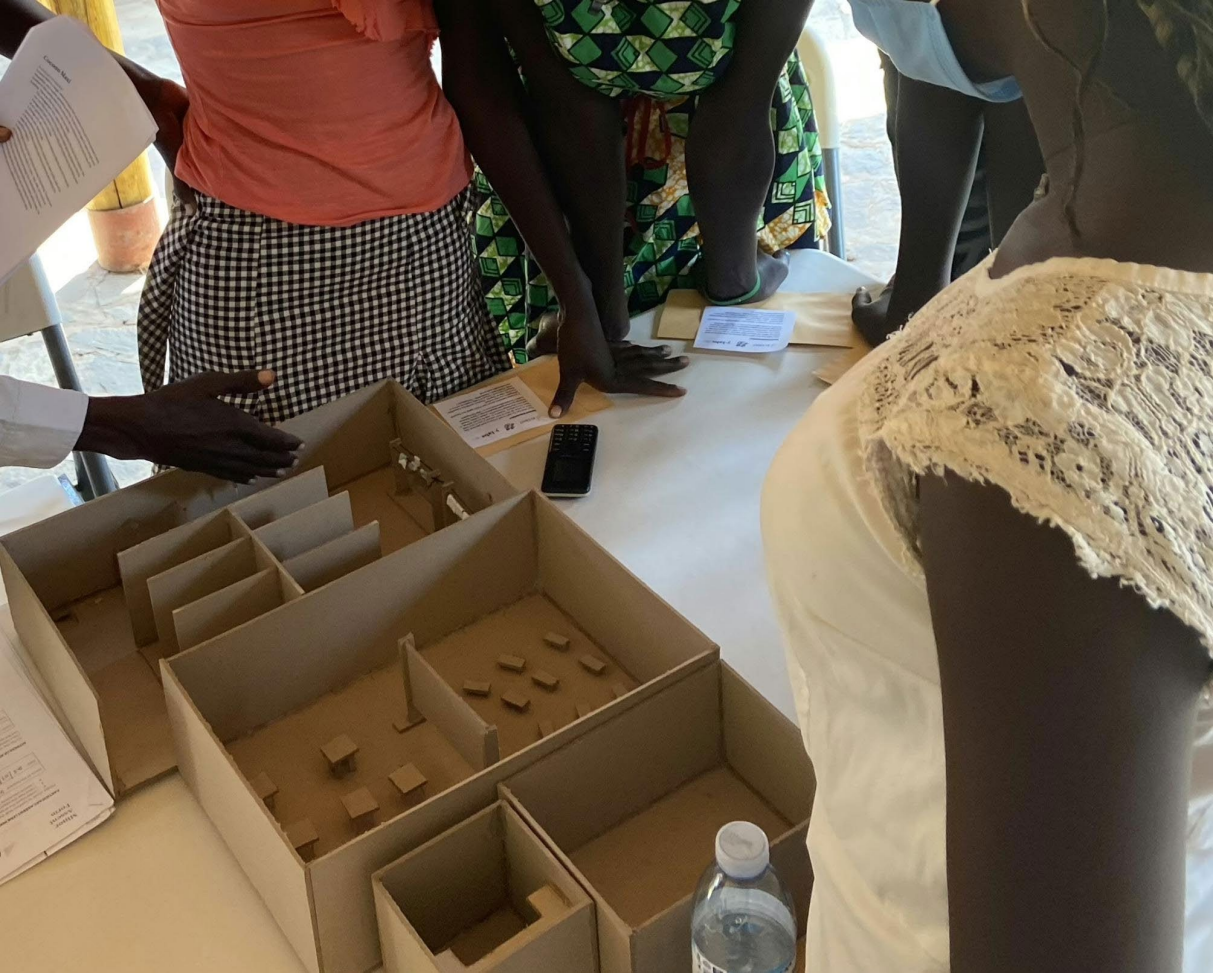
A rough prototype of a safe, public space for MH

Based on the findings from design research, four rough prototypes were created and tested to address both hardware (i.e. physical spaces and materials) and software (i.e. education and sensitization) solution components. The purpose of rough prototyping is to test essential questions about the desirability and feasibility of early solution concepts with minimal materials. We use rough prototypes to learn directly from our users about how they would change the idea in order to quickly iterate and improve potential solutions. In working groups, we invited all of the same participants from the design research phase to provide input on these prototypes, given their comfort speaking about this sensitive subject matter and their familiarity with the project. Physical models, illustrations, and voice recordings were used to help participants immerse themselves in the features and scale of the prototypes (Fig. 2) and provide feedback on their desirability, cultural acceptability, and feasibility.

### Phase four: live prototyping

Once these ideas were refined based on interview feedback, the most promising concepts determined by the highest desirability, cultural acceptability, and feasibility were advanced into live prototyping. Live prototyping seeks to test high-fidelity prototypes of materials and products in a real-life setting [27]. We invited 86 new individuals, which included 58 people who menstruate, 13 boys, and 15 health and humanitarian workers, to engage with high-resolution prototypes, use them over several weeks, and give feedback to the design team for further improvement. During the three-week live prototyping period, participants took part in a pad-making enterprise and used the first prototype of a physical space for managing menstruation. We analyzed the supply chain for materials to make both pads and physical structures, spoke with stakeholders on the feasibility of procurement and building, and asked participants under what conditions they would use these spaces and services, and how important they were to daily life.

### Phase five: pilot

The prototype with the highest desirability for use expressed by participants, and feasibility for implementation by settlement stakeholders and community leaders, was tested during a three-month pilot from May to July 2021. The prototype, called a Cocoon Mini (Mini), is a safe and private MH space that provides access to a private latrine, bathing facility, and product disposal options.

A total of 20 Minis were constructed in May 2021 in Ariwa I and Ariwa II, which are located approximately 10 km apart. Minis were built in homestead compounds, rather than public areas, to allow for more privacy during use. Homestead compounds are defined as a given piece of land on which a family or family cluster constructs their homes. Each Mini directly served 15–20 people who menstruate residing in the chosen homestead, though the facility was available for all people who menstruate in the community at large to use. Homesteads were chosen using three criteria: first, they are located at least 200 m from each other to ensure equitable distribution of Minis across the community; second, they are located at least 100 m from a pre-existing main water source to provide easier access to those without convenient water access; and third, they were built near respected community members who could serve as supervisors and ensure that the space was adequately maintained. Supervisors were also tasked with overseeing the cleaning of the Minis, reporting any concerns that arose throughout the pilot period, and educating community members on the purpose of the Mini.

We conducted structured interviews with 109 people who menstruate with Minis in their homesteads, 64 other community members (both men and women) without Minis in their homesteads, and all 20 Mini supervisors. Throughout the pilot, Mini supervisors were interviewed twice, one to two months apart, to determine if the spaces had incurred damage over time. Kuja Kuja research team members living in the community employed convenience sampling to recruit people who menstruate who had used the spaces and were available to participate and provide feedback on usability. A simple random sampling of community members without Minis in their homesteads, and living at least 100 m away from a homestead with a Mini, was used to gauge community acceptance of the space.

A qualitative interview guide was developed for each of the three types of participants in order to elicit information about their overall experience using the Cocoon Mini or their opinions of the space to gauge acceptability. A range of structured questions were included to see what participants liked and disliked about the space, the Mini’s impact on their daily lives, and any concerns they may have about integrating the space into their community. Additionally, two open-ended questions were further quantified into frequencies with percentages to assess the total number of people who menstruate who reported that the Mini has made MH easier for them and which Mini features had the most impact. Supervisors were asked questions about cleaning and maintaining the Mini space. Interviews were conducted in English, Bari, or Juba Arabic by members of the Kuja Kuja team at participants’ homes or a location of their choosing. All interviews were transcribed and translated into English by the interviewer who conducted them. Subsequently, a second interviewer verified the completion of the interview and the accuracy of the translation from Bari or Juba Arabic to English to ensure the interview’s validity. Throughout the project, COVID-19 protection measures were taken to ensure the safety of participants and research team members.

Interviews were translated into English and de-identified transcripts were uploaded into a secure site for data management. Transcripts were then analyzed using a blended approach of deductive and inductive coding thematic analysis to identify and explore patterns of meaning across the interviews [28, 29]. The research team developed an initial thematic coding framework based on the structured questions from the interview guide, specifically to assess the Mini’s desirability, acceptability, sustainability, and potential for impact. The team coded all transcripts and modified the scheme using the initial coding structures until no new codes were identified.

YLabs team members met through a series of remote meetings with the Kuja Kuja field team for further validation, discussion, and consensus of the results. The research team also took additional open-ended field notes throughout the building process and pilot to assess implementation and maintenance feasibility. These notes included informal discussions and observations that were incorporated into the analysis.

We obtained written and verbal informed consent for all participants. In the case of minors, both informed assent and parental consent were obtained. The study protocol was reviewed and approved by the Research and Ethics Committee of Makerere University of Public Health in Kampala, Uganda.

## Results

### Phase one: background research

Stakeholders within the settlement spoke of exposed, crowded toilet and bathing facilities in schools and in the community. Using these facilities, especially at night, often left girls and women vulnerable to sexual and gender-based violence [30]. Long-lasting water shortages, coupled with scarcely available clean water and limited access to soap, made bathing a challenge. Pad procurement and cleaning also proved challenging. From our expert interviews, we learned that people who menstruate typically rely on humanitarian organizations for access to new pads; when supply chains are disrupted, they are likely to switch to homemade cloth pads or re-use pads before they are fully dry, which often leads to infection. Water and soap shortages force people who menstruate to wear pads longer than intended or dispose of reusable pads sooner.

Product disposal is particularly challenging, as it has not historically been part of the MH infrastructure in humanitarian settings. Evidence from the literature demonstrates the importance of discreet disposal solutions on people who menstruate’s psychological well-being, ability to stay in school or at work, and the overall impact on environmental toxicity and chemical pollution [31–36]. Without formal disposal options, people who menstruate are forced to venture out to discard sanitary products in forest areas, open grounds, or bodies of water, away from the public eye, and sometimes before sunrise or after sunset [1, 30]. This puts them at risk for violence [37]. Safer but potentially incomplete disposal options, such as pit latrines, may leave them vulnerable to shaming and verbal harassment if their materials are found by others in the community. People who menstruate also shared a deep fear of used sanitary products being found and used for witchcraft [1, 2, 38].

### Phase two: design research

Insights generated during design research with residents of the settlement aligned with three topic areas: information and education, products and supplies, and supportive physical infrastructure.

First, participants explained that sexual education focused predominantly on abstaining from sex to avoid pregnancy, leaving little room for discussion on menstruation, puberty, and bodily autonomy. During interviews, girls asked questions regarding the physiology of menstruation, how to stay clean and manage pain during menstruation, and the risk of pregnancy during the menstrual cycle. Men and boys shared that they are largely excluded from conversations on MH, which fuels rumors and narratives around menstruation being ‘dirty’, and that those who are currently menstruating should not participate in certain activities.

*“Girls should not cook while menstruating because, if men know, some of them will not want to eat your food.” - Participant, age 16*

Second, the supply chain of sanitary pads into the settlement has been severely disrupted with the withdrawal of humanitarian organizations due to the COVID-19 pandemic. This has forced people who menstruate to sell food rations in order to afford sanitary pads. People who menstruate may also re-use pads immediately after washing before they have dried, or use makeshift materials in place of pads, both of which can lead to infection.

*“I buy the pads from the market. If there’s no money to buy sanitary pads, I sell off food rations to get the money.” - Participant, age 19*

*“Here, [humanitarian] partners give disposable pads. And once they are done, you have none.” - Participant, age 18*

Finally, girls articulated that existing spaces for managing menstruation are not suiting their needs. Public areas for MH are clearly marked as such, making their menstruation status conspicuous to the community. Attendants control both the spaces and the provision of MH products and often deny girls access or privacy, making these spaces unsafe for use. This, in turn, may cause anxiety and limit physical mobility, as there are few suitable places outside the home to manage menstruation.

*“There are girls who have faced harassment because they went [to the public MH space] without getting permission from the attendant. The attendant chased them away and they did not get any help.” - Participant, age 17*

### Phase three: rough prototyping

Based on these insights and topic areas, four prototypes were developed to address challenges related to education, menstrual products, and supportive infrastructure.

**Fig. 3:**
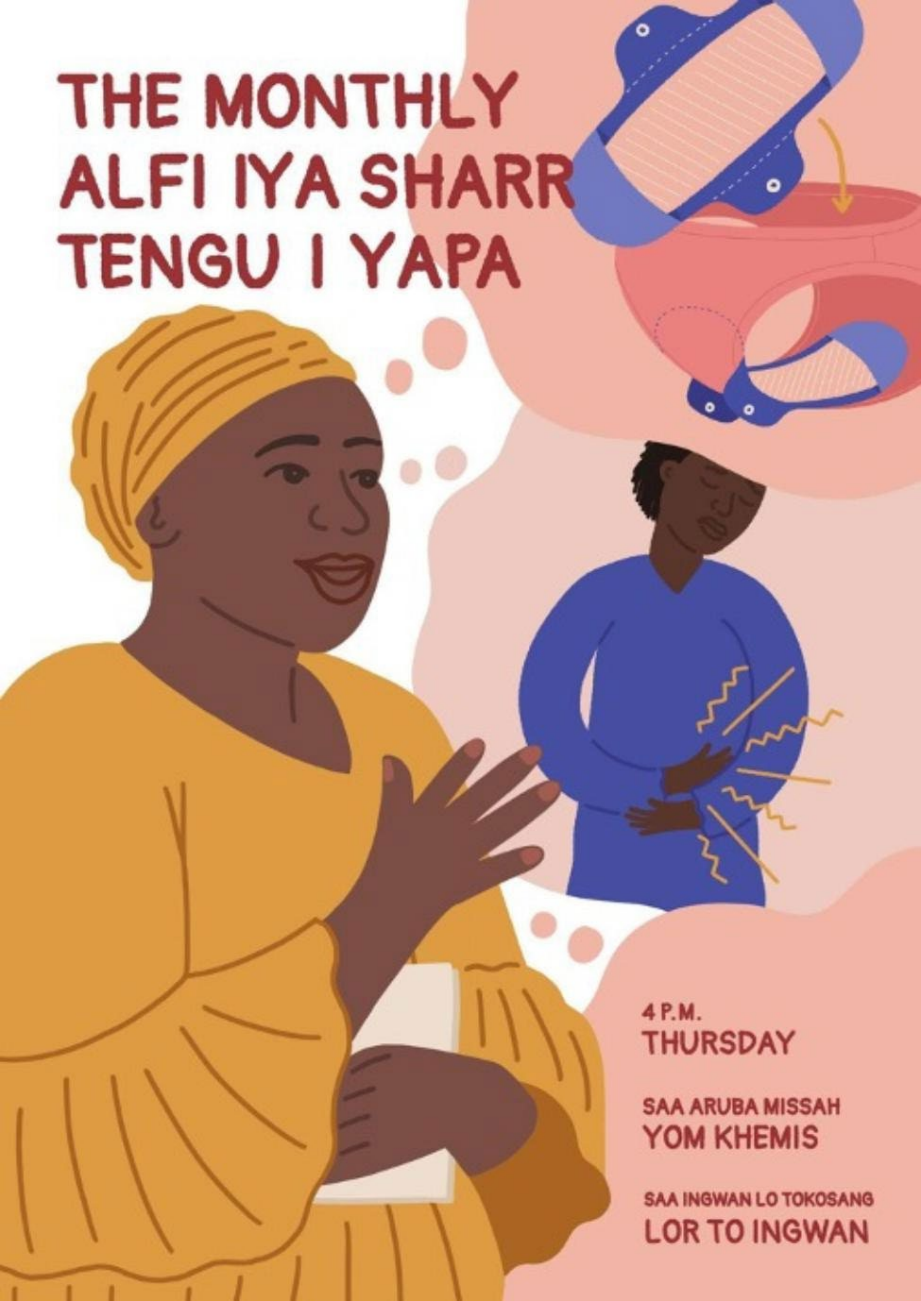
Poster for monthly storytelling event

To generate conversation and increase education about menstruation, we tested the idea of a monthly community storytelling event (Fig. 3). During the event, young women and girls would share their experiences with menstruation and sexual health, along with educational content and sanitary product demonstrations. Female participants felt comfortable sharing their own stories in this space, but believed men should have a separate conversation to learn about menstruation that is facilitated by a health worker or older male figure with accurate information. Furthermore, such an event would require an ongoing investment in both compensation for attendance and sensitization efforts with parents and husbands of participants to garner full community support. Ultimately, we were not able to take this idea forward due to emerging COVID-19 restrictions limiting community gatherings at that time.

**Fig. 4:**
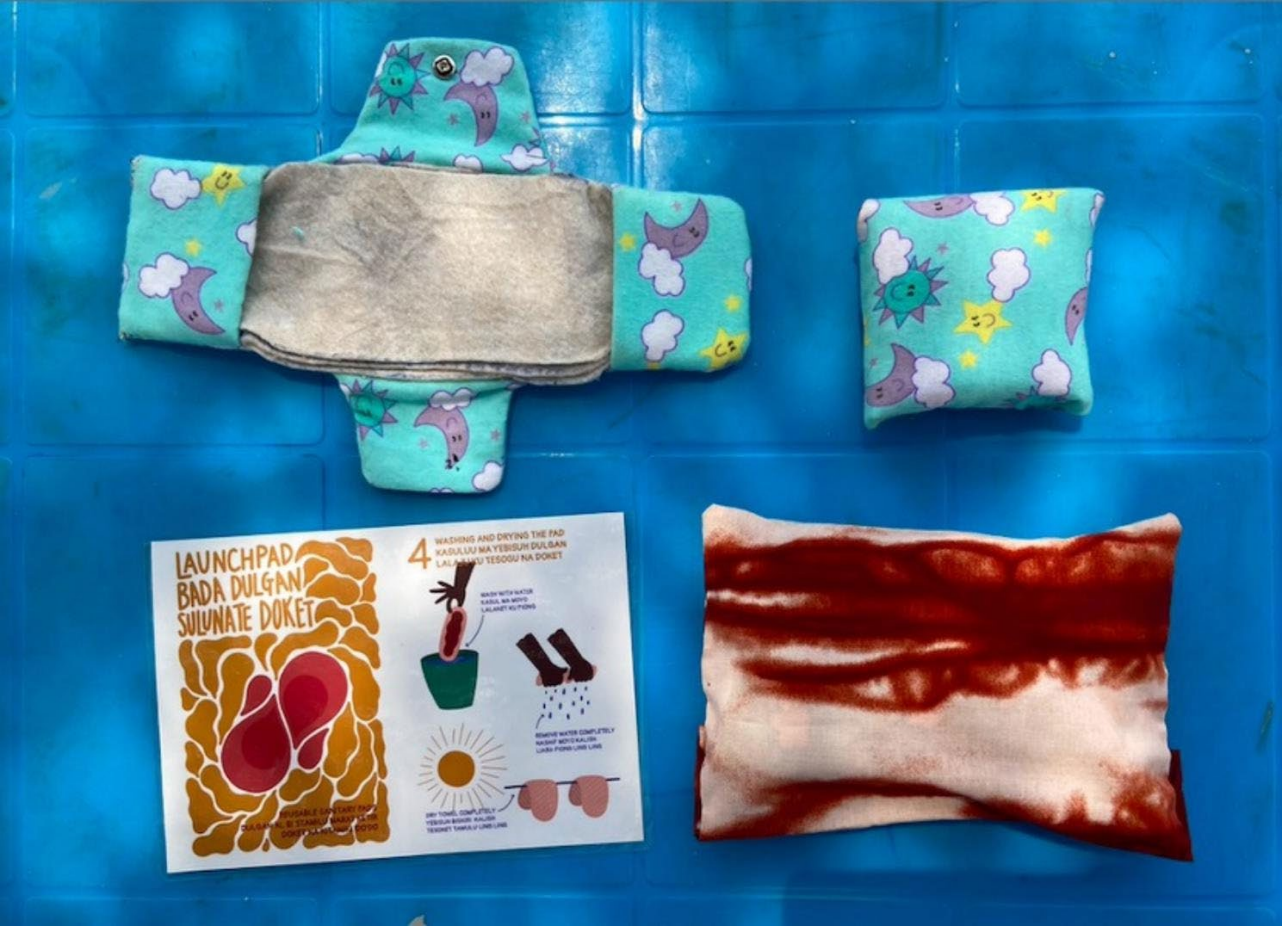
LaunchPad sanitary pad, carrying case, and instructional pamphlet

To increase access to sanitary products and supplies, we created the LaunchPad, a community-run social enterprise that would hire and upskill people who menstruate to make reusable pads (Fig. 4). The pads would then be sold to other people who menstruate in the community, allowing those involved to turn a small profit and develop new skills. Participants liked the idea of sustaining their own demand for sanitary products but still felt it necessary to partner with humanitarian non-governmental organizations (NGOs) to ensure the quality of the product, cost subsidies, and more robust supply chains. We advanced this idea to live prototyping to explore this further.

To provide more supportive physical infrastructure for managing menstruation, we tested the idea of both a large community space (the Cocoon Maxi) and a smaller homestead space (the Cocoon Mini). The Maxi was intended to serve as a multi-purpose center that offered programs and services, especially for young women and girls, peer group support, and access to secure latrines. However, in speaking with community stakeholders, we learned that securing construction materials and approval for the land from the government and humanitarian actors could take six months or longer. The Mini, on the other hand, faced fewer logistical barriers to construction given its smaller footprint and location within homesteads, making this idea more feasible.

### Phase four: live prototyping

Based on their feasibility and the interest they generated during rough prototyping, the LaunchPad and the Cocoon Mini advanced to the live prototyping phase with higher-fidelity mockups of these ideas.

Testing for The LaunchPad began with a workshop for tailors in the community to create reusable pads and carrying cases. These were then distributed to a group of young women and girls along with an instructional pamphlet that illustrated steps on how to wear, wash, and dry the pads. We surveyed all participants over the course of three weeks and found that, although desirability for the program was high, girls were concerned about maintaining their reusable pads without regular access to water and spaces for drying. Furthermore, because of fluctuations in pad distribution by NGOs, the supply and demand of pads remain inconsistent in the settlement, which could compromise the business model of uninterrupted sales. Ultimately, the sustainability of the program could not be confirmed, and we were unable to advance this prototype to the pilot phase.

Next, we tested the Cocoon Mini in a live setting. Three Mini structures were constructed with privacy walls, locks on doors, and solar lights, in accordance with user feedback from rough prototyping. As a result, the majority of participants reported an improved sense of safety. Water access and washing areas provided a strong motivation to use the space, as they made bathing and cleaning reusable pads more convenient. Participants articulated the importance of local ownership in the construction and maintenance of the site in order to foster a sense of communal responsibility.

*“I can now bathe three times a day when menstruating because of easy access to water and the privacy of the bathroom. The one we had before had holes in it, so I was not able to bathe during the day due to fear that people may see me.” - Participant, age 15*

Participants also noted a lack of discreet product disposal options. The most commonly used option is a pit latrine, which does not completely dispose of sanitary products and often collapses when overfilled. Participants like that incinerators allow for complete disposal, but do not feel comfortable carrying their sanitary products to current burn sites, which are often far away. Many participants requested an incinerator be attached to each Mini, though this posed logistical and financial challenges. Two incinerators were therefore constructed alongside one Mini structure in either village to reduce travel time to existing incinerators.

*“Girls and women have refused to take the waste products to be burned at the current site of the incinerator in [another village]. They said they are feeling ashamed to carry and even collect because it looks unhygienic. - Mini Supervisor*

### Phase five: pilot

Due to its reported improved sense of safety and feasibility for construction, the Mini prototype was selected for a three-month pilot program. Construction materials, including iron and plastic sheets, timber, cement, and pipes, were procured in nearby Yumbe town. Metal frames and doors for drying bins were also fabricated in Yumbe. A local hardware shop owner served as foreman for the project by mobilizing a team in Yumbe for materials transport to the settlement. Each of the 20 Mini cost approximately $360 USD in materials and labor. Each Mini served 15–20 people who menstruate, leading to a cost per person of $18–24 USD. The two incinerators installed cost an additional $2110 each.

Male community members were recruited and paid to aid professional plumbers in digging and backfilling pipe trenches. Later, they also assisted masons in building the latrines by framing sites, laying foundations and bricks, digging drainage trenches, and constructing walls. Men stated that they appreciated the opportunity to learn construction skills, receive compensation, and have more water access points. Furthermore, their involvement increased exposure to and acceptance of the idea of a designated menstrual health space.

**Fig. 5:**
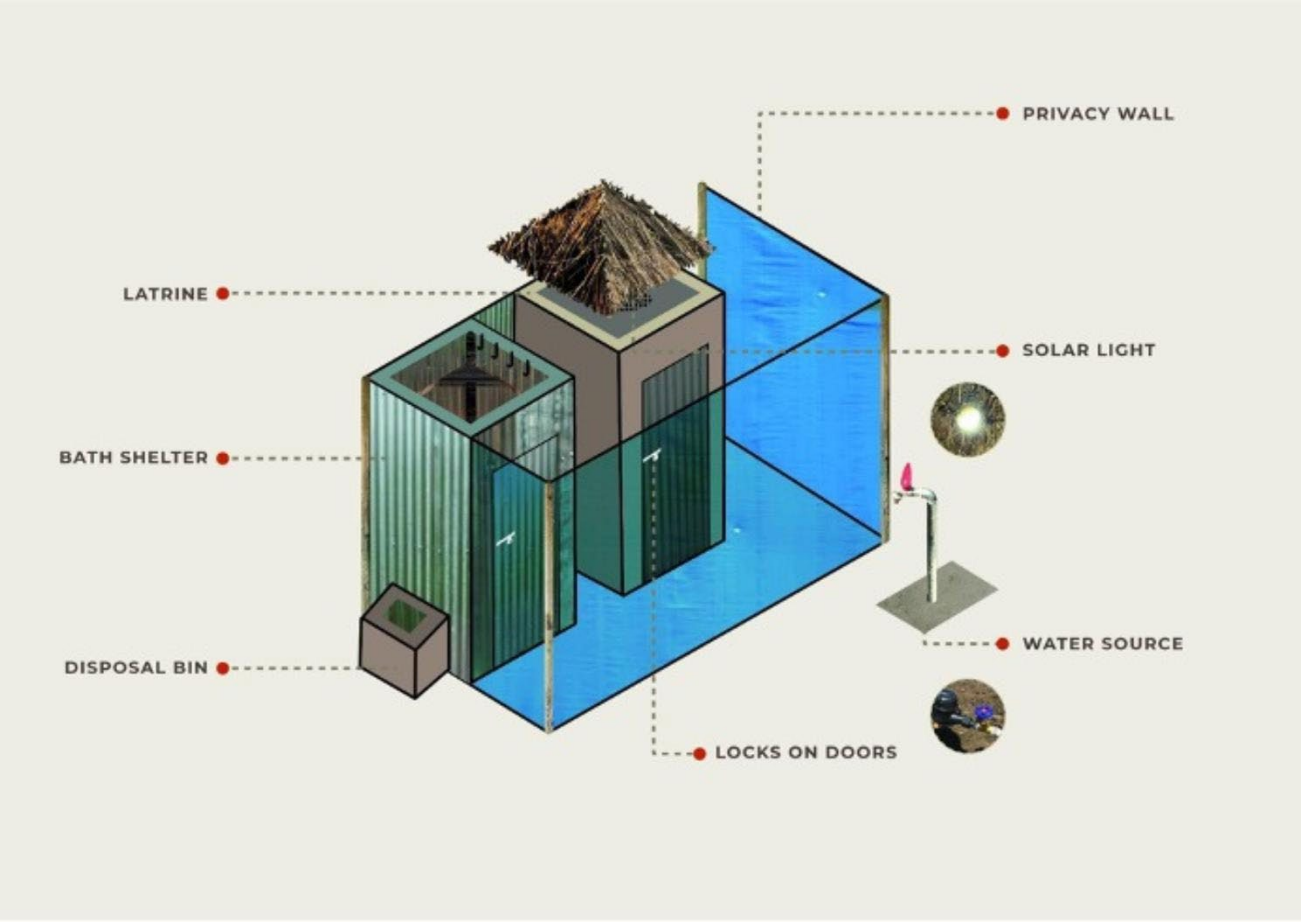
Rendering of the Cocoon Mini highlighting key features

**Fig. 6:**
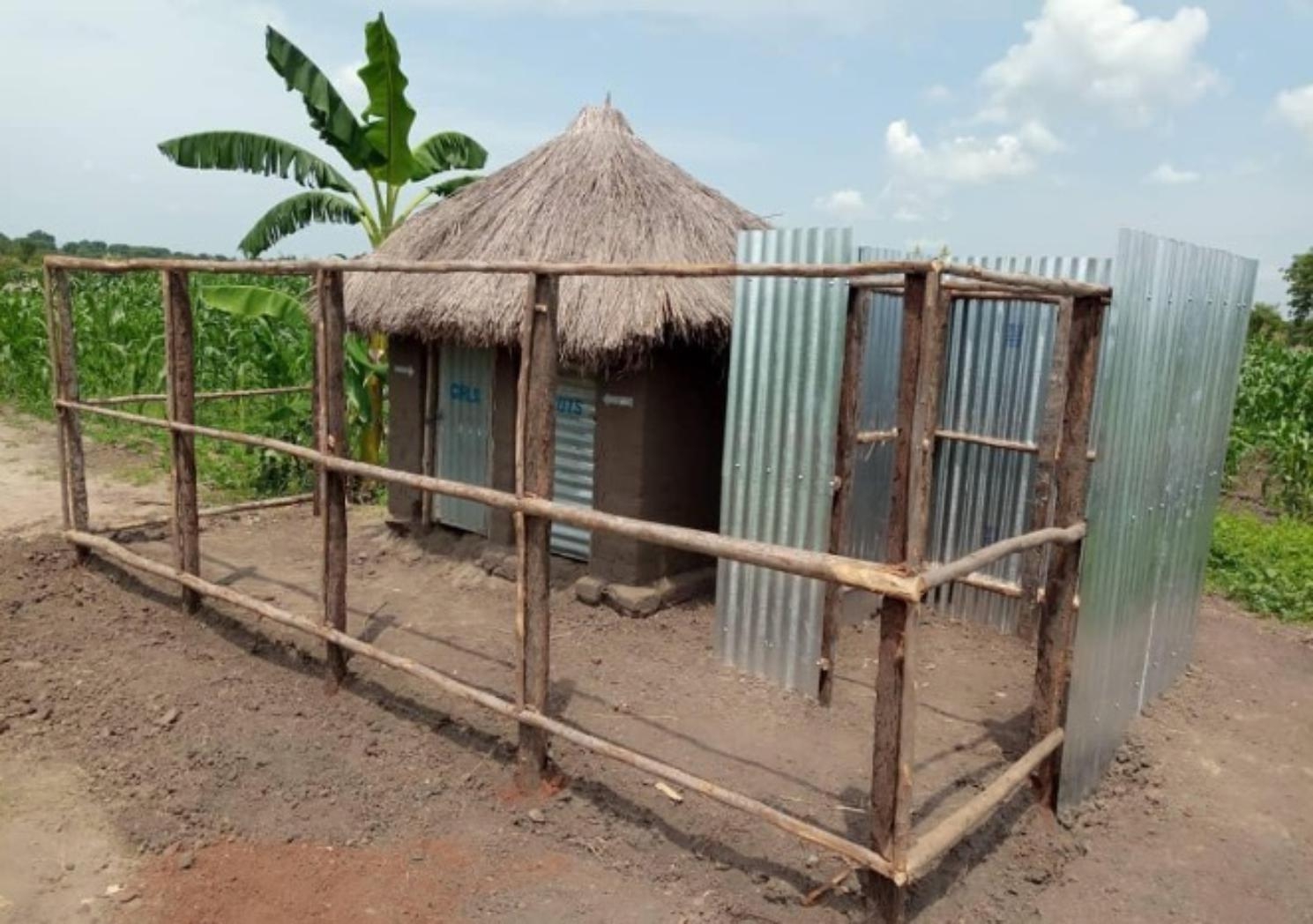
A built Cocoon Mini in the Bidi Bidi Settlement

Each structure was built alongside a pre-existing pit latrine by attaching a bathing structure made out of iron sheets. All Minis incorporate a 1.7-meter vertical high privacy wall constructed from plastic tarps that span the length of both the latrine and bath shelter to increase users’ privacy (Figs. 5 and 6). In addition, each Mini was outfitted with locks on all the doors, portable solar lights, and a disposal bin. An additional water source tap was also built next to every Mini for ease of washing sanitary products and bathing access. Covered drainage pipes were provided to redirect wastewater away from the bath shelter. In both Ariwa I and II, one Mini was fitted with an incinerator to assist with product disposal once the waste bins were full.

A total of 193 individuals were interviewed during the pilot, including 109 people who menstruate with direct access to a Mini within their homestead, 64 other community members without a Mini in their homestead, and all 20 Mini supervisors (Table 1).

**Table 1:** Study Sample Characteristics

Variable	Mini Users from Selected Homesteads (n = 109)	Community Members (n = 64)	Mini Supervisors (n = 20)
Age 15–18 19–24 Above 24	41 (37.6%) 39 (35.8%) 29 (26.6%)	24 (37.5%) 30 (46.9%) 10 (15.6%)	0 (0%) 0 (0%) 20 (100%)
Sex Female Male	109 (100%) 0 (0%)	52 (81.3%) 12 (18.7%)	6 (30%) 14 (70%)
Area Arwia I Arwia II	51 (46.8%) 58 (53.2%)	28 (43.6%) 36 (56.3%)	9 (45%) 11 (55%)

### Achievements

Achievements of the pilot study are highlighted in Table 2. Overall, the 20 Cocoon Minis served an estimated 300 people who menstruate throughout the pilot, and showed high desirability and acceptability among people who menstruate, community members, and supervisors. Overall, 94% (102/109) of people who menstruate expressed that they approved of the Cocoon Mini space. Supervisors and community members also had positive reactions to the Mini as they relayed the importance of having a space dedicated for MH.

*“Most people appreciate having the Mini in the community because it has improved the hygiene and sanitation of girls.” - Participant*, *age 24*

*“I would be willing to [to build a Mini] if I would have money because it is helping girls, families, and those women and girls in the neighborhood”* - *Male community member*

The Minis improved water access and introduced a designated waste disposal system, making MH easier and more accessible for people who menstruate. Overall, 95% (104/109) of people who menstruate with a Mini in their homestead stated the space has made menstrual hygiene management easier for them. When people who menstruate were asked an open-ended question on which Mini features made MH easier to manage, nearly all participants cited water access (96%), followed by the disposal system (47%). Lighting within and surrounding the MH space was another feature that was highly valued, as it allowed people who menstruate to use the Mini at night.

*“TheMini has made MH easier because bathing is possible now with access to water, a disposal bin, and light to use at night hours.”* - *Participant, age 31*

The added water tap access provided people who menstruate with the flexibility to bathe more frequently and at more hours of the day or night at their convenience. People who menstruate reported improved hygiene practices and decreased feelings of physical discomfort from not wearing products longer than intended. Having regular and dependable access to the Mini’s water source increased people who menstruate’s confidence in consistently and adequately managing their menstruation while participating in daily activities.

*“There is change in my daily life because we now have excess water that can allow us to maintain personal hygiene during menstrual flow.” - Participant, age 22*

The designated disposal bins were also a critical component of the Minis, as they reduced users’ stress of seeking a place for disposal and allowed them to change and dispose of their used products more frequently. Regularly, people who menstruate cited no longer needing to throw away their used materials in the bush or look for safe places for disposal. However, they would only use the disposal bins after installing a padlock to the bin at each site. The padlock served as a vital component of the utility of the disposal bin, giving people who menstruate a sense of peace knowing that their disposable pads would not be tampered with by others in the community.

*“Girls are scared of disposing of used pads in the bin on assumption that kids will open and spread them around, as it is not locked.”*- *Mini Supervisor(before padlock was added)*

*“The Cocoon Mini has helped me because I no longer dispose of used pads in the bush as usual since the Mini spaces are in the villages.” - Participant*, *age 26(after padlock was added)*

Results indicated how a physical space dedicated to MH can have an effect not only on improving people who menstruate’s hygiene practices, but also influence their physical and psychological safety. During the three-month pilot, there were no reported safety incidents from people who menstruate, community members, or supervisors. The Mini was seen as a space where menstrual status could easily be concealed from boys and men, so they were no longer easy targets of ridicule or belittlement. The Mini’s high privacy walls, locking doors, and lights improved people who menstruate sense of privacy and security from possible intrusion.

*“The privacy wall, doors, and locks make me feel safe because they offer protection while using the space.”* - *Participant, age 15*

*“I like the Mini because it’s private. No one can know or see you inside.”* - *Participant, age 26*

Before the Minis were built, people who menstruate reported being restricted to their home during menstruation, as access to essential changing and bathing facilities or bathrooms was lacking within the community. Strategically placing Minis throughout the community improved people who menstruate’s autonomy and spatial mobility, as they could freely move within the community to markets, health centers, and schools because they could confidently access any available Mini.

*“When I am going to school I can access any Mini within the community to bathe and change rather than coming home.”* - *Participant, age 21*

*“[The Mini] made it easier because when I’m going to the market, I can change in any Mini space within the community.”* - *Participant, age 21*

People who menstruate would often have to sacrifice significant time and energy during the days of their active cycle in order to accommodate their flow, including fetching water or searching for disposal or washing facilities. By having water access closer to bathing areas, people who menstruate can now save valuable time and energy that they otherwise would spend by traveling long distances to fetch water and waiting in long lines.

*“I no longer go to fetch water from far, but rather go to bathe and change within the Mini.”- Participant, age 17*

*“Sometimes our bathroom may be very busy, but now I can just go to bathe in the Mini and go to school or the market”- Participant, age 21*

Furthermore, by creating a safe, dedicated household space for menstruation, participants reported speaking more openly about MH after observing and actively participating in MH best practices. In total, 47% of people who menstruate reported having more discussions about MH with men and boys since the creation of the Mini space. Having a physical space for men and boys to ask about, and for people who menstruate to directly refer to, made them feel more comfortable and confident talking about MH to other community members. They liked explaining the importance of the Mini features.

*“Those days, I used to fear talking about menstruation, but since the creation of this space, it became easier to explain to men about periods.” - Participant, age 20*

*“[The Mini] has made me learn more, especially about bathing frequently during menstruation, which I could also talk about to other people [like men].”- Participant, age 18*

**Table 2:** Summary of Achievements

Achievement	Description
Widespread desirability and acceptability of the Mini	The Mini was highly desired by people who menstruate and community members due to its safety features and water accessibility. The Mini was also the first-ever physical space dedicated specifically to helping people who menstruate manage their menstruation in this community.
Improved menstrual hygiene management practices	The design features of the Mini, including the waterline, disposal system, and lights, contributed to improved MH practices. People who menstruate could now change, bathe, and wash and dispose of their products more frequently.
Increased physical and psychological safety	The privacy of the Mini gave people who menstruate an increased sense of physical and psychological safety. People who menstruate reported reduced anxiety in knowing where and when they could manage their menstruation without the threat of intrusion.
Autonomy and spatial mobility	The accessibility of the Minis provided people who menstruate with the flexibility and capability to manage their menstruation at their convenience. People who menstruate were also able to freely move within the community, knowing they had multiple spaces to manage their menstruation instead of having to return home to do so.
De-stigmatization of menstruation	The Minis often sparked conversations with men and boys, allowing for open dialogue about menstruation to occur, often for the first time.

### Challenges and Learnings

The pilot study highlighted several operational challenges that could impact the feasibility of the intervention if scaled (Table 3). Heavy rains in the settlement delayed work, as the settlement’s roads are of poor quality, and vehicles run the risk of getting stuck when they flood. This led to a delay in material delivery. Additionally, the project’s timeline was heavily impacted by events that occurred within the settlement, and by the COVID-19 pandemic. We recommend considering external events that might increase construction time, as anything from food distribution schedules to rain will halt all progress on construction.

The Minis were built to be as safe and sturdy as possible while also complying with policies set forth by the Office of the Prime Minister, which discouraged the use of permanent structures. Still, it is important to note that people who menstruate and community members desired structural permanence. Temporary or makeshift structures are prevalent in the settlement and are prone to rapid degradation; however, psychologically, the permanence of a space imparts value, especially for communities that have experienced much transition. Thus, it is crucial to people who menstruate that the spaces they use are durable and long-lasting, and many requested that stronger and more durable materials be used. Over the three-month pilot, a small number of supervisors and people who menstruate reported minor damage and deterioration to the Mini space from weather and termites.

*“The materials are plastic sheets, iron sheets, timber, and cement. I don’t like the structure because it’s temporary. Next time, let them make a permanent structure with its latrine such that everyone can feel free to use and it lasts longer.” - Participant, age 21*

*“Tarpaulin at the privacy walls are tearing off [from strong winds] and termites have started eating the joining poles.” - Mini Supervisor*

Although people who menstruate appreciated the addition of a disposal bin to hold used products, an incinerator at each Mini would have ensured the complete destruction of products. Spaces that offer disposal options need to take into account that people who menstruate will not have peace of mind unless their discarded products cannot be uncovered, seen, or tampered with. Literature and evidence from the field show that people who menstruate prefer to have an incinerator on-site so they are not required to carry their product from the Mini to a separate incinerator when the disposal bin is full. Most people who menstruate were afraid of being seen carrying used menstrual products to the incinerator site and being teased by men and boys.

.*“I would change the idea of having a bin and rather install an incinerator so that waste can be burnt within, since girls fear to collect the waste product.” - Participant, age 15*

*“Girls and women refuse to collect the waste products to be burned at the site where the incinerator is. They said that they have fear and shame of carrying the waste so boys or other people may laugh at them. Let them rather have incinerators at all the Cocoon Mini sites so waste can be easily burned.”- Mini Supervisor*

Although the Minis provided a safe, private space for people who menstruate, we found that the structures still require free supplies in order to be fully functional, as not all people who menstruate owned necessary MH materials. Many people who menstruate reported that they or their peers do not have the funds to supply their own pads and soap and thus felt excluded from using the Mini. Many people who menstruate requested that all Minis should be supplied with basic MH supplies. When asked an open-ended question on which MH materials Mini should include, the top three answers were soap (56%), buckets (46%), and sanitary pads (38%). While providing MH supplies was out of the financial scope of this study, future projects may benefit from including these commodities.

*“Is there any plan of delivering items like soap, pads, knickers (underwear), and buckets at the Minis to support us? We do not have money for purchasing them from markets.”- Female community member, age 19*

While the Mini served as a hardware solution by offering a physical space for MH, it was notably lacking a direct software component. Menstruation is still associated with feelings of shame, sexualization, and uncleanliness in many communities. Despite data showing that the Mini made it easier to talk about menstruation more openly, nearly all people who menstruate reported that they would still not feel safe with boys and men using or being around the Mini, for fear that they would humiliate them or expose their menstrual status to the public. This fear led to some people who menstruate not using the space.

*“It’s in our culture that men and boys are not supposed to see or know anything about menstruation unless married [couples] are to share among themselves.” - Participant, age 19*

*“[I don’t use the Mini] because I have fear that people from outside may laugh at me since the space is inside their compound.” - Female community member, age 23*

*“Nothing would make me feel safe around menstrual hygiene management with boys and men because I fear they might expose me in public.” - Female community member, age 24*

People who menstruate also reported feelings of fear, shame, and embarrassment when they had to walk with buckets and soap to and from the Mini, and other community members would see their supplies.

*“My mother does not use the Mini because she feels ashamed to move with buckets, underwear, pads, and soaps at the neighborhood’s compound.”- Participant, age 19*

Furthermore, we found there was a lack of accurate information around sexual/reproductive health and MH among people who menstruate. A pervasive fear in the settlement was that sexually transmitted infections (STIs) could be contracted from using the communal Mini space due to hygiene concerns amid its increased usage.

*“My sister doesn’t use the space because many people from different households are using it and she is fearing that she may get infected [with STIs].”- Participant, age 26*

**Table 3:** Summary of Challenges

Challenge	Description
Operational and logistical barriers	Procurement of materials, impassable roads, and unplanned settlement events impacted the project’s timeline.
Structural permanence	Psychologically, structural permanence imparts value. It is essential to people who menstruate that the Minis are durable and built with materials to withstand wear and tear, which requires a larger financial investment.
Inadequate disposal options	Spaces that offer disposal options need to consider that people who menstruate will not have peace of mind unless their discarded products are safe from tampering and are destroyed at the disposal site.
Lack of MH supplies	Not all people who menstruate who visit the Minis have the necessary materials for maintaining their hygiene, and this makes them feel excluded from using the Mini.
Lack of software solution to complement hardware solution	Sensitization around MH and educational SRH forums for both boys and girls are needed in order to prevent stigmatization and misinformation around MH, STIs, and general hygiene.

## Discussion

This is the first study to apply HCD principles to co-design and create a community-driven solution of safe spaces for menstrual health management among a displaced population. The Cocoon Mini served as a proof of concept of the utility of applying HCD methodology in co-designing new MH interventions with people who menstruate. The Coon Mini also demonstrated that such a space could largely be run and maintained on the local level without the need for continued heavy stakeholder engagement. Overall, the pilot project was widely accepted by people who menstruate and other community members and resulted in great improvements in people who menstruate’s confidence. Furthermore, the Minis helped alleviate the psychosocial stress people who menstruate experienced using unsafe spaces, and increased their autonomy and spatial mobility by providing easily accessible changing, washing, and disposal spaces throughout the community.

This research identified five impactful components of menstrual health spaces: disposal and drying bins, lighting within and surrounding the MH space, privacy walls, locks, and water access, building upon previous evidence [38]. Along with these features, people who menstruate also desired that their menstrual health space be gender-segregated and have at-source disposal technology, ideally an incinerator. These components can be replicated in other contexts after being customized for cultural and infrastructural considerations, and will also be critical to the scaling and future adaptations of Mini spaces. Community buy-in and ownership of the Mini were also fostered by involving local men in the construction of the Mini [1].

While the physical structure and design attributes of the Mini played a pivotal role in ensuring people who menstruate’s safe experience, the stigma around menstruation was still pronounced in the community. Our findings indicate that a more holistic approach including both hardware and software solutions is vital to ensure lasting change. Potential software solutions to accompany implementation of hardware solutions like the Cocoon Mini include social and behavioral interventions that foster dialog and challenge traditional beliefs. To ensure long-term, lasting change, targeted sensitization forums where comprehensive sexual/reproductive health issues can be openly discussed by men and women alike are necessary [39]. With open dialogue and sensitization training, men and boys can understand menstruation and, ideally, gradually become advocates for the well-being of the women and girls in their community [40].

The Cocoon Mini has the potential to expand far beyond the bounds of the Bidi Bidi Settlement with the scalability of the physical spaces interlinked with the sustainability of the solution itself. Using HCD to understand each community’s specific needs would allow for nuanced customization of the Minis based on cultural beliefs and available infrastructure. We found that the iterative, creative, and user-centered nature of the HCD process allowed the team to work in partnership with both people who menstruate and key community stakeholders to prototype and develop solutions which integrate people who menstruate’s priority needs for safety, privacy, hygiene, and disposal. Furthermore, local ownership of Mini construction and maintenance allowed for agency and decision-making power to be concentrated within the community rather than with NGO partners or project staff.

### Limitations

There are certain limitations of the pilot that are important to note. First, Kuja Kuja staff, who are members of the Bidi Bidi community, recruited participants, conducted interviews, and transcribed answers into English. Though the familiarity between interviewers and participants likely provided a more comfortable atmosphere for interviews, we recognize that it may have also introduced social desirability bias into the study. Second, the Mini users recruited for this study were chosen from a convenience sample, and the sample size was limited by available resources and time constraints. We recognize that data may not be representative of all members of the community. Third, pilot data collection was conducted only over a short three-month period, with some interviews taking place early on after the Mini was built, limiting our ability to assess long-term changes. Finally, the Bidi Bidi Settlement is well-established and has some pre-existing infrastructure; therefore, our study findings and implementation strategy may not be generalizable to other humanitarian settings that are more emergent.

### Future research

There are further opportunities to research the widespread effects of the Minis. Our data suggested that a dedicated space to privately manage menstruation may improve safety for people who menstruate; longer-term research should explore if the Minis could ultimately play a role in reducing gender-based violence within the settlement. Secondly, our qualitative findings indicate that the use of Minis could potentially reduce school/work absenteeism. A more formal testing of this hypothesis could provide valuable insight into understanding the wider impact of safe MH spaces. Lastly, the HCD approach in designing the Minis with the support of the Bidi Bidi community members presents a co-design methodology for future projects seeking to center community engagement and capacity-building, potentially decreasing reliance on NGOs and aid partners, while promoting skill-building and independence in humanitarian contexts.

## Conclusions

Limited access to safe, private spaces for MH is a pressing problem for people who menstruate in humanitarian crises, as the design of current sanitation facilities does not address the challenges of menstruation. The Cocoon Mini, co-designed with a refugee population, is a promising option for people who menstruate to be able to manage their menstruation safely and effectively, and potentially have a meaningful impact in their lives beyond MH, for a relatively small financial investment. Therefore, MH facilities like the Cocoon Mini should be considered a high-priority intervention in humanitarian settings.

## Data Availability

The datasets used and/or analyzed during the current study is available. from the corresponding author on reasonable request.

## List of Abbreviations

HCD: Human-centered design
LMICs: Low and middle-income countries
MH: Menstrual health
NGO: Non-governmental organizations
STIs: Sexually transmitted infections
WASH: Water, sanitation, and hygiene
YLabs: Youth Development Labs
